# Breast cancer adaptive resistance: HER2 and cancer stem cell repopulation in a heterogeneous tumor society

**DOI:** 10.1007/s00432-013-1494-1

**Published:** 2013-08-30

**Authors:** Nadire Duru, Demet Candas, Guochun Jiang, Jian Jian Li

**Affiliations:** 1 Center of Excellence in Translational Human Stem Cell Research, California National Primate Research Center, University of California Davis, Sacramento, CA 95817 USA; 2 Department of Radiation Oncology, University of California Davis, Sacramento, CA 95817 USA; 3 Department of Medical Immunology and Microbiology, University of California Davis, Sacramento, CA 95817 USA; 4 NCI-Designated Comprehensive Cancer Center, University of California Davis, Sacramento, CA 95817 USA

**Keywords:** Tumor-acquired resistance, NF-κB, HER2, STAT3, Breast cancer stem cells

## Abstract

**Purpose:**

The lethal effects of cancer are associated with the enhanced tumor aggressiveness in recurrent and metastatic lesions that show resistant phenotype to anti-cancer therapy, a major barrier to improving overall survival of cancer patients. The presence of heterogeneous populations of cancer cells within a specific tumor including the tumor-initiating cells or so-called cancer stem cells (CSCs) has linked the acquired resistance (AR, or adaptive resistance). Herein, we discuss the CSC-mediated tumor repopulation in AR of breast cancer in this review.

**Methods:**

We emphasize a dynamic feature of gene induction in tumor cells that undergo long-term treatment, and describe a specific HER2-NF-κB-HER2 pro-survival pathway that can be initiated in breast CSCs upon radiation therapy.

**Results:**

Elucidation of HER2-induced pro-survival networks, specifically the force driving tumor repopulation due to radioresistant CSCs during anticancer therapies, will have a significant impact on the generation of new diagnostic and therapeutic targets to control of recurrent and metastatic breast tumors.

## Introduction

The recurrence and metastasis of primary tumor cells to distant organs, particularly to bone, lungs, liver, and brain following traditional anti-cancer therapy have been the challenge for successful anti-cancer strategies for breast cancer (Lacroix 2006). Radiotherapy continues to be a powerful tool for the control of tumor growth, contributing to the overall survival rate of the majority of cancer patients. However, it is well known that tumors develop adaptive response (AR) to radiation therapy and become more resistant, aggressive, and invasive (Ahmed and Li 2007). Revealing the insights of the acquired radioresistance in the recurrent and metastatic tumors is essential to further improve the efficacy of cancer treatment.

Cancer stem cells (CSCs) definition emerged shortly after the discovery of only a small fraction of tumor cells being able to form colonies or new tumors (Al-Hajj et al. 2003; Bonnet and Dick 1997). These fractions express specific cell surface markers that differentiate them from the rest of the tumor cell populations. The initial stem cell markers of breast cancer are defined as CD44^+^/CD24^−/low^ (Al-Hajj et al. 2003). Cells with these cell surface markers are shown to have elevated levels of pro-invasive genes required for metastasis, including interleukins such as IL-1α, IL-6, IL-8, and urokinase plasminogen activator (UPA) (Sheridan et al. 2006). However, further studies suggested that CD44^+^/CD24^−/low^ feature alone is not enough for the spread of breast cancer (Sheridan et al. 2006) pointing to the existence of additional markers to define potential breast CSCs. In 2007, Ginestier et al. introduced aldehyde dehydrogenase-1 (ALDH1) as an additional marker for the breast CSCs, and recently, our laboratory showed that HER2 is a novel breast CSC marker responsible for their resistance to radiation therapy (Duru et al. 2012). The continuous and urgent need to define specific biomarkers to precisely identify breast CSCs for the early detection of tumor progression and the prevention of metastasis accelerated the research in this area extensively.

Clinical data suggest that breast cancer patients with tumors overexpressing HER2/neu, a member of ErbB family of receptor tyrosine kinases (RTKs), live one-third shorter than the patients with a HER-2/neu negative tumor, and the enhancement of *HER2* copies is also correlated with the relapsing time of the disease (Arteaga et al. 2012; Asada et al. 2002; Slamon et al. 1987). We have previously shown that, upon radiation exposure, HER2 activates a pro-survival transcription factor, NF-κB, through Akt-mediated pro-survival pathways (Guo et al. 2004), and interestingly, *HER2*, itself, is among the genes that are transcriptionally activated by NF-κB upon radiation, indicating a positive feedback loop between HER2 and NF-κB (Cao et al. 2009). Defining the central role of NF-κB in these pathways may offer new therapeutic targets for breast cancer treatment (Ahmed et al. 2006); however, it also increased the challenge of implementation since NF-κB is currently involved in the regulation of more than 150 target genes (Pahl 1999). Therefore, detecting HER2 as both downstream and upstream element of NF-κB signaling is encouraging given that this unique relationship may allow novel approaches to target HER2 in breast cancer therapy. In this review, we will discuss recent findings in pro-survival signaling networks that are critical for the acquired tumor resistance in breast CSCs.

## Tumor adaptive response, CSC-mediated tumor cell repopulation

Increased tumorigenicity of an identifiable subpopulation of cancer cells with specific surface markers was first studied in acute myeloid leukemia (AML), where these cells were referred to as cancer stem cells (Bonnet and Dick 1997; Pardal et al. 2003). CSC concept supports a hierarchical organization of the tumor cells and predicts that only a specific subset of tumor cells with stem cell-like properties, such as the capacity to self-renew and generate the heterogeneous lineages of cancer cells, is able to initiate tumorigenesis. Several CSC markers have been reported for a variety of solid tumors including breast, brain, prostate, pancreas, and colon cancers (Hermann et al. 2007; Hurt et al. 2008; Patrawala et al. 2006; Ricci-Vitiani et al. 2007; Singh et al. 2004). The finding that only a small subset in entire tumor cells has this significant self-renewal potential and the ability to proliferate in an uncontrolled manner (Al-Hajj 2007; Al-Hajj et al. 2003) challenged the assumption that the chance of each cancer cell to form a new tumor is equivalent (O’Brien et al. 2010). These features in CSCs render them a hot topic in cancer research, especially since new evidence emerges frequently to support their roles in tumor recurrence, aggressiveness, and therapy resistance, which results in inefficient cancer treatments (Duru et al. 2012; Gangopadhyay et al. 2013; McDermott and Wicha 2010).

Radiotherapy has been extensively used for tumor control via the initiation of DNA damage-induced cell death. However, long-term observations of irradiated cells have revealed that apart from apoptosis, a variety of cell fates emerge among irradiated cell populations (Forrester et al. 2000; Prieur-Carrillo et al. 2003), suggesting that specific mechanisms were activated in the surviving heterogenic population of tumor cells. Therapeutic efficiency of ionizing radiation (IR) is associated with IR-induced apoptotic responses (Almasan 2000; Dewey et al. 1995), genomic instability (Morgan 2003; Morgan and Murnane 1995), bystander effects (Klokov et al. 2004; Morgan 2003; Morgan and Murnane 1995), and adaptive radioresistance (Ch’ang et al. 2005; McBride et al. 2002). Radioresistance is also linked with the signaling cascades activated during temporary but significant cell cycle arrest (Bebien et al. 2003; Fornace et al. 1999; Hartwell and Kastan 1994), the degree of DNA damage, the activation of signaling networks; and the activation of pro-survival or pro-apoptotic signaling pathways determines the fate of an irradiated cell (Feinendegen 1999, 2002; Maity et al. 1997; Schmidt-Ullrich et al. 2000; Stecca and Gerber 1998; Waldman et al. 1997; Weichselbaum et al. 1994; Wolff 1989, 1998). The induced protection/tolerance of irradiated cells is also evident since pre-exposure to a low or intermediate dose of X- or γ-rays reduces the lethal effects and genomic instability caused by subsequent exposures of higher doses of IR (Kelsey et al. 1991; Olivieri et al. 1984; Robson et al. 2000; Skov 1999; Suzuki et al. 1998, 2001). Therefore, a better understanding of the molecular mechanisms underlying the tumor adaptive response is necessary to further increase the efficacy of anti-cancer therapy.

The tumor radioresistance creates a serious challenge to the current cancer treatments (Stockler et al. 2000), and CSCs are shown to be more radioresistant than the non-stem cancer cells, suggesting that the decrease in the tumor size after radiotherapy results mainly from the sensitivity of non-CSC cancer cells rather than a previously believed random tumor cell death (Baumann et al. 2008). In addition to their resistant phenotype, Al-Hajj et al. (2003) showed that CSCs are more tumorigenic than non-stem cancer cells, such that breast cancer cells expressing CD44 (CD44^+^) but not CD24 (CD24^−/low^) are more tumorigenic since as few as 100 cells with this phenotype are able to form tumors in mice while millions of cells missing this phenotype are not. This resulted in the identification of CD44^+^/CD24^−/low^ as the marker of breast CSCs in 2003 (Al-Hajj et al. 2003). Thereafter, Phillips et al. (2006) showed that the breast cancer cells expressing CD44^+^/CD24^−/low^ are more radioresistant, supporting a notion that CSCs are more radioresistant than the non-stem cancer cells and placing CSC research at the core of tumor adaptive radioresistance studies.

CSCs are believed to be responsible for treatment failure and tumor recurrence (Al-Hajj et al. 2004; Reya et al. 2001). Repopulation of cancer cells upon anti-cancer therapies has long been considered as the cause of treatment failure (Kim and Tannock 2005). Dylla et al. (2008) showed the repopulation and increased tumorigenicity of colorectal CSCs (CD44^+^ESA^+^) in xenogeneic tumors subsequent to chemotherapy (Dylla et al. 2008). In a recent paper, Pajonk’s group showed that IR is capable of reprogramming differentiated breast cancer cells into induced breast CSCs (iBCSCs). Moreover, iBCSCs displayed enhanced mammosphere formation and tumorigenicity as well as expressed the same stemness-related genes as BCSCs from non-irradiated samples. Their study proposed that BCSC phenotype was induced by radiation in differentiated breast cancer cells, which contributed to the enrichment of BCSCs after anti-cancer treatments (Lagadec et al. 2012). Repopulation of CSCs has also been supported clinically, such that the percentages of CSCs are found to be increased following cytotoxic chemotherapy in breast cancer patients (Diehn et al. 2009). Diehn et al. (2009) discussed that it is plausible to assume the presence of different subclones of CSCs with different sets of mutations/genomic alterations within tumors, since heterogeneous tumors consist of unstable genomes. Upon chemo- or radio-therapy, the CSC clones with the advantageous genomic alteration to protect against therapy would be selected for and continue to sustain the tumor (Diehn et al. 2009). To support this idea, Bao et al. (2006a, b) reported that glioma cancer stem cells acquired radioresistance by promoting DNA damage repair (Bao et al. 2006b). Odoux et al.’s (2008) study supported the existence of genomic instability in the CSCs showing that the karyotypes of parental tumor cells were mostly similar to the derived clones except some of them had unique chromosomal aberrations (Odoux et al. 2008). It is also possible that during the progress of cancer, anti-cancer therapy might cause the CSCs to acquire new mutations that might render CSCs more resistant to therapy as well as helping them self-renew. This theory might also explain the higher percentages of CSCs seen in the recurrent tumors (Diehn et al. 2009). Klonisch et al. concluded that CSCs can appear as a result of modifications in the microenvironment of the stem cell niche, as a result of mutations that might lead to a change in the signaling pathways and cell cycle control or as a result of enrichment of cell populations with specific phenotypes (Klonisch et al. 2008). Li and Neaves (2006) proposed that the differences between normal stem cells and CSCs are the degree of their dependence on the specialized microenvironment of stem cells, so-called stem cell niche, and they discuss that an alteration in the signaling networks governing the homeostatic regulation of the niche might disrupt the stem cell maintenance. Therefore, CSCs may arise from an intrinsic mutation leading to self-sufficient cell proliferation or alteration of the niche by proliferation-promoting signals that might become dominant. Liu and Wicha (2010) reinforced the importance of stem cell niche for the regulation of cellular functions. They discuss how breast CSCs are governed by the elements that form stem cell niche, in addition to the intrinsic signals. Their review brings up the importance of paracrine interactions in the regulation of cellular functions and emphasizes that some of these altered interactions are involved the signaling pathways, which include Wnt, Notch, and Hedgehog (Liu and Wicha 2010).

These findings shed light onto a new conceptual paradigm of how breast CSCs or cancer-initiating cells contribute to the radiation response. There are numerous complicated mechanisms involved in adaptive resistance. It is realistic to think that radiation-induced mutations in some tumor cells give survival advantage to the cells and contribute to their acquired radioresistance. However, it is also likely that radiation selectively kills the relatively radiosensitive tumor cell populations leaving the therapy-resistant CSCs alive, thus contributing to adaptive radioresistance via the selective repopulation of CSCs. Our recent publication showing the repopulation of HER2^+^ breast CSCs upon radiation treatment provides another evidence that supports the latter (Duru et al. 2012). Further studies are necessary to fully understand the exact mechanisms of the acquired radioresistance, but the involvement of selection along with the introduction of mutations seems crucial. The elucidation of the key features of the adaptive response is essential for the discovery of specific gene/protein targets to re-sensitize radioresistant fractions of the tumor that will ultimately increase the efficacy of anti-cancer therapy.

## DNA repair and the radioresistance of CSCs

One of the common characteristics of normal stem cells and CSCs is their ability to protect DNA from stress-induced damage substantially better than the non-stem cells (Frosina 2009). The average mutation rate in somatic cells in vivo is 10^−4^, as opposed to a 10^−6^ in embryonic stem cells (Hong et al. 2007), suggesting an enhanced DNA repair activity in stem cells. Although radiation induces multitudes of damage in cells, the main mechanism of radiation-mediated killing is via generating hard-to-repair DNA damage after apoptosis (Bao et al. 2006a). Understanding the detailed mechanisms by which stem cells retain less DNA damage is crucial for the treatment for cancers resistant to radio- and chemo-therapy.

Increased ability of CSCs for DNA repair (Johannessen et al. 2008) accounts for the low rate of apoptosis they have. Bao et al. (2006a) reported that glioma stem cells are able to promote radioresistance by enhancing DNA damage repair and lowering the rate of apoptosis. Their results indicate the radiation-induced repopulation of CD133^+^ tumor cells, which are suggested as glioblastoma CSCs (Liu et al. 2006). CD133^+^ glioma cells survive radiation by preferentially activating DNA damage checkpoints and repairing the radiation-induced DNA damage more effectively than the CD133^−^ glioma cells. This was supported by the findings that the addition of inhibitors specific for Chk1 and Chk2 checkpoint kinases sensitized the radioresistant CD133^+^ glioma cells (Bao et al. 2006a). Consistent with it, Rich (2007) proposed that the transient activation of the DNA checkpoints leads to cell cycle arrest, which is a required step for the initiation of DNA repair process. Accumulating evidence demonstrates a relationship between radioresistance and DNA damage signaling via a variety of mechanisms (Puc et al. 2005; Skvortsova 2008). It is of great value to clarify whether these mechanisms are predominantly activated in the CSCs compared to other tumor cell populations. Skvortsova et al. (2008) suggested that the radioresistance in prostate carcinoma cells is achieved by the activation of signaling pathways controlling cell survival and DNA repair. Phosphoglycerate kinase 1 (PGK1) is one of the proteins up-regulated in the radioresistant prostate cancer cell lines, which is known to have roles in both DNA replication and repair in mammalian cells. Moreover, PGK1 was identified as a downstream effector of HER2 signaling and contributes to the aggressiveness of the breast cancer. Another DNA base damage repair protein up-regulated in radioresistant prostate cancer cells is DNA-(apurinic or apyrimidinic site) lyase, which is encoded by the *APEX1* gene and normally involved in the repair of pre-mutagenic lesions. It was shown to mediate DNA damage repair via the regulation of several transcription factors including NF-κB (Skvortsova 2008). Induction of NF-κB has also been associated with the loss of PTEN, a tumor suppressor gene that negatively regulates Akt signaling pathway (Chu and Tarnawski 2004). Interestingly, the induction occurs via PI3K/Akt pathway, suggesting a positive feedback mechanism, which is suggested to be involved in the cancer chemoresistance (Gu et al. 2004). In addition to PI3K/Akt pathway, other signaling pathways including Ras/MAPK induced by several cytokines, growth factors, and tyrosine kinases can also activate NF-κB.

NF-κB activation is a transient process that has to be tightly regulated to avoid overenhancing the survival of the cells. In tumor cells, dysregulation of different signaling pathways as well as alterations in the activity or the expression of several genes may lead to the misregulation of NF-κB, enabling its constitutive activation. These genes are involved in cell cycle control, migration, adhesion, and apoptosis among the NF-κB target genes (Dolcet et al. 2005). Lavon et al. (2007) reported one of the first data showing the role of NF-κB in the regulation of DNA repair mechanisms. O^6^-methylguanine-DNA methyltransferase (MGMT) is a DNA repair enzyme, which is responsible for the resistance of cancer cells to several alkylating agents, thus conferring chemoresistance to certain tumor types (Lavon et al. 2007; Margison et al. 2003). The elevated activity of MGMT has been detected in many types of tumors including breast cancer, although the levels of activation were variable and even absent in some tumors (Margison et al. 2003). In glioma cell lines, the activity of NF-κB is associated with the expression of MGMT (Lavon et al. 2007). Further experiments showed that NF-κB is a major player in the regulation of MGMT, suggesting a new model for the mechanism of DNA damage repair mediated by NF-κB upon exposure to alkylating agents (Lavon et al. 2007). Accordingly, it is plausible to suggest a link between the activation of DNA damage proteins and NF-κB-HER2-NF-κB feedback loop in radioresistant breast CSCs. As a matter of fact, the relationship between NF-κB activity and radioresistance has been shown in MCF7 breast cancer cells (Cao et al. 2009). Further studies are crucial to demonstrate that this relation is exclusive to BCSCs and might contribute significantly to their radioresistance. Moreover, the conceivable model of Lavon et al. (2007) points to new targets for developing therapeutic strategy to cure chemo-resistant tumors. To support this, our most recent data further suggest that large sets of DNA repair proteins were up-regulated in HER2+ BCSCs (Duru et al. 2012). We believe that, in the near future, studies focusing on the relation between DNA damage response and therapy resistance in CSCs will result in the development of new therapeutics against radioresistance.

## Pro-survival signaling networks in CSCs

Therapeutic IR causes DNA damage and generates oxidative stress in cells leading to the activation of specific signaling pathways in the irradiated cells (Spitz et al. 2004). Depending on the extent of DNA damage, either pro-apoptotic or pro-survival pathways are initiated. Studies on glioma CSCs revealed the complex regulation of CSCs. Several signaling pathways including the activation of RTKs, bone morphogenetic proteins (BMPs), Hedgehog, and Notch are shown to be important for governing glioma CSCs. The epidermal growth factor receptor (EGFR), a member of RTK family, is shown to play a significant role for the proliferation and neurosphere formation in glioma CSCs. Activation of pro-survival PI3K/Akt pathway, which is downstream of RTKs, has been shown to be more dominant in glioma CSCs compared to non-stem glioma cells. Hedgehog pathway that is also reported to be active in gliomas has been suggested to be required for self-renewal of CSCs (Li et al. 2009).

Hambardzumyan et al. (2006) showed the importance of the activation of PI3K/Akt/mTOR pathway in conferring radioresistance to subpopulations of medulloblastoma cells. They showed that at least three different populations of tumor cells give different responses upon IR. The main cell population, which was radiosensitive, underwent p53-dependent apoptosis. The other two populations were radioresistant, including nestin-expressing stem cells and non-proliferating cells. The nestin-expressing stem cells underwent p53-dependent cell cycle arrest, while the non-proliferating cells did not respond to radiation (Hambardzumyan et al. 2008). Phillips et al. (2006) showed that Notch signaling pathway is activated in breast CSCs through PI3 K pathway upon exposure to radiation, which resulted in increased number of CSCs (Phillips et al. 2006). Notch1 has been shown to be overexpressed in breast cancer, and its expression levels have been associated with breast cancer prognosis. Several genes, including HER2, CyclinD1, CDK2, and Notch4, are up-regulated via activation of Notch1 signaling (Phillips et al. 2006). The importance of Notch pathway in breast cancer stem cells has been previously shown, and the Notch-binding sequence was found in HER2 promoter, suggesting a relationship between Notch and HER2. Moreover, HER2-overexpressing cells show active Notch signaling (Korkaya and Wicha 2009). Interestingly, the formation of iBCSCs and thus the enrichment of BCSCs upon anti-cancer treatment can be prevented to some extent with the inhibition of Notch signaling pathway, suggesting that targeting Notch signaling might be a good treatment strategy after radiation therapy (Lagadec et al. 2012). Several other studies also point to the importance of targeting Notch signaling pathway as an anti-cancer treatment. The role of Notch1-mediated signaling pathway in maintenance of neural stem cells, which contributes to growth and progression of glioblastomas, is essential, and it has been shown that Notch1 receptor promotes survival of glioblastoma cells by regulation of the anti-apoptotic Mcl-1 protein, which is mediated by the induction of EGFR (Fassl et al. 2012; Wang et al. 2010). Clementz et al. showed that Notch-1 and Notch-4 are novel targets of PEA3 in breast cancer cells and suggested that targeting PEA3 and Notch signaling pathways would provide a new therapeutic strategy for triple-negative and possibly other breast cancer subtypes (Clementz et al. 2011). A significant association between the increased expression of Notch1 and HER2 in breast cancer suggested that Notch signaling pathway should be a therapeutic target, especially for HER2-positive breast cancers with poor prognosis (Zardawi et al. 2010).

## NF-κB initiated pro-survival networks

NF-κB-mediated pro-survival pathway is one of the major signaling pathways activated by DNA damage (Ahmed and Li 2008). NF-κB is a sequence-specific transcription factor originally involved in inflammation and carcinogenesis (Baldwin 1996; Karin 2006). In typical/canonical pathway, the components of the prototypical NF-κB transcription factor, p65 and p50, form a heterodimer that remains inactive in the cytoplasm in association with its inhibitor, IκB. The phosphorylation, dissociation, and proteolysis of IκB are mediated by the IκB kinase (IKK) complex, which contains two catalytic subunits, IKKα/IKK1 and IKKβ/IKK2, and a regulatory subunit IKKγ/NEMO (NF-κB essential modulator). Upon release of IκB, NF-κB is free to translocate from cytoplasm to the nucleus to regulate the expression of its target genes (Granville et al. 2000; Lenardo and Baltimore 1989; Li and Verma 2002). Besides its role in carcinogenesis, NF-κB is shown to prevent apoptosis in transformed cells and enhance survival in many types of cancers (Baldwin 2001; Danial and Korsmeyer 2004; Gilmore 2003; Jung et al. 1995; Kataoka et al. 2002; Kucharczak et al. 2003; Tang et al. 2001). Not surprisingly, accumulating evidence indicates that NF-κB and its controlled pro-survival elements play an essential function in the response of cells to low or high doses of IR (Ahmed et al. 2009; Brach et al. 1991; Luo et al. 2005). Recent data demonstrate that IR can activate NF-κB through an atypical/non-canonical pathway that is initiated in the nucleus via ATM-mediated SUMO (small ubiquitin-like modifier) pathway that involves the sumoylation of NEMO in the cytoplasm, which then results in its nuclear translocation and subsequent association with ATM in the nucleus. ATM-dependent phosphorylation causes the nuclear export of NEMO and activation of the typical pathway (Curry et al. 1999). This is an important finding that established the mechanism of the activation of cytoplasmic stress sensors like NF-κB by DNA damage signals that are predominant in the nucleus of an irradiated cell.

Many studies, some still ongoing, investigate the effects of the inhibition of NF-κB activity in the radiation response. Inhibition of NF-κB has been shown to modulate ATM-associated apoptosis (Jung and Dritschilo 2001; Jung et al. 1997) and notably to enhance heat-mediated radiosensitization (Curry et al. 1999; Locke et al. 2002). However, blocking NF-κB activity has been shown to result in a variant degree of sensitization of tumor cells to therapeutic radiation. This inconsistency highly likely stems from the ability of NF-κB to regulate many different effector genes involved in a wide array of physiological functions, sometimes even causing opposite effects (Barkett and Gilmore 1999; Romashkova and Makarov 1999). Therefore, it is crucial to further identify and therapeutically target the specific NF-κB effector genes that mediate the survival response upon IR, especially those functioning in tumor-acquired radioresistance and/or radiation-resistant cancer stem cells. NF-κB signaling network was shown to be activated in breast CSCs (Diehn et al. 2009). Our studies demonstrated that NF-κB can be activated via HER2 overexpression, and active NF-κB, subsequently, causes increased overexpression of HER2 in breast cancer cells with the radioresistant phenotype (Cao et al. 2009). This study indicated the existence of an elegant survival strategy used specifically by breast CSCs. Our results support the hypothesis that HER2-NF-κB-HER2 loop is specifically activated as a survival mechanism in the breast CSCs upon radiation treatment, which may confer radioresistance to CSCs and will be discussed in details below.

## Overexpression of HER-2 in breast cancer and therapy resistance

In clinic, *HER2* is overexpressed in 25–30 % of the total breast cancer patients (Haffty et al. 1996; Valabrega et al. 2007). HER2 overexpression is found to be associated with aggressive tumor growth, resistance to treatment, metastasis, and a high risk of local relapse and recurrence resulting in poor prognosis (Haffty et al. 1996; Holbro et al. 2003; Slamon et al. 1987). HER2, which is linked to BCSCs (Diehn et al. 2009), was found to be a crucial biomarker for many types of cancer based on its expression levels despite a lack of gene mutation or truncation of the protein (Warren and Landgraf 2006). Furthermore, HER2 expression level is being used as a predictive marker for the diagnosis of metastatic breast cancer and is an important factor for designing a treatment plan (Haffty et al. 1996; Hicks et al. 2005).

HER2 is a proto-oncogene located in the long arm of human chromosome 17 and encodes a 185 kD transmembrane glycoprotein in tissues of epithelial, mesenchymal, and neural origin (Olayioye 2001; Soomro et al. 1991). HER2 belongs to the ErbB family of receptor tyrosine kinases, which comprises four members: EGFR (ErB1/HER1), ErbB2/HER2/Neu, ErbB3/HER3, and ErbB4/HER4 (Citri and Yarden 2006). Signal transduction from these receptors is initiated by ligand binding to the extracellular domain of the receptor followed by receptor dimerization and trans-autophosphorylation of specific tyrosine residues within the receptor’s cytoplasmic domain. The phosphorylated active receptor then recruits downstream signaling proteins containing Src homology 2 (SH2) and phosphotyrosine binding (PTB) domains with a high affinity for phosphotyrosine residues. The binding of these specific effector proteins to the activated receptor leads to the activation of many different signaling pathways including Ras-MAPK, phosphatidylinositol 3’kinase-protein kinase B (PI3K-PKB/Akt), and phospholipase C–protein kinase C (PLC-PKC) pathways and enables receptor coupling to biological responses (Warren and Landgraf 2006). Although there have not been any soluble ligands identified for HER2 so far, it plays a crucial role in ErbB signaling via its strong kinase activity and as the preferred partner of other ErbB family members (Citri and Yarden 2006; Warren and Landgraf 2006). Moreover, HER2 can spontaneously form homodimers and automatically phosphorylate itself to obtain intrinsic tyrosine kinase activity (Eccles 2001). Not surprisingly, altered ErbB signaling has been involved in cancer development and progression since it is responsible for regulating proliferation, survival, and/or differentiation (Britten 2004; Warren and Landgraf 2006).

Studies on ErbB family members and their involvement in cancer development and progression led scientists to develop antibodies against some individual members of the family. The anti-HER2 monoclonal antibody, rhumAbHER2 (trastuzumab/Herceptin), is the first of these anti-cancer agents suppressing HER2 activity (Uno et al. 2001). It is the humanized form of the murine 4D5 antibody that is directed to the external domain of HER2 and inhibits the growth of the cells that are positive in HER2 expression. FDA approved this anti-HER2 antibody to be used in treatment for metastatic breast cancers overexpressing HER2 (Liang et al. 2003b; Slamon et al. 2001). Herceptin, which has been shown to inhibit proliferation of the breast cancer cells, also promotes the radiation-induced apoptosis and radiosensitize the cancer cells depending on their level of HER2 expression (Liang et al. 2003b). Both primary and metastatic breast tumors have been shown to have overexpression of HER2 (Valabrega et al. 2007), suggesting the potential success of Herceptin as a cure for both primary and metastatic breast cancer. Clinical trials show, however, that patients with HER2 protein overexpression (detected by immunohistochemistry (IHC)) are less likely to respond to the Herceptin treatment than patients with amplified HER2 gene copy number detected by fluorescence in situ hybridization (FISH) (Mass et al. 2005). So far, the only explanation given for this inconsistency is the different sensitivities of the IHC and FISH methods in determining the HER2 protein levels and gene copy numbers, respectively (Press et al. 2002; Valabrega et al. 2007). However, we propose an alternative explanation to this controversy in the following section, involving several factors, mainly HER2 and NF-κB, which have important functions in major signaling pathways.

HER2 overexpression induces mammary carcinogenesis, tumor growth, and invasion affecting normal and malignant mammary stem cells (Korkaya et al. 2008). The number of the stem/progenitor cells increases in normal mammary epithelial cells upon overexpression of HER2. The mammary cells with enhanced HER2 levels also increased ALDH1 levels (Diehn et al. 2009). These data are significant given that ALDH1 is suggested as a CSC marker for some tumors, including breast cancer (Diehn et al. 2009; Ginestier et al. 2007).

## NF-κB-regulated HER2 gene expression

**Fig. 1:**
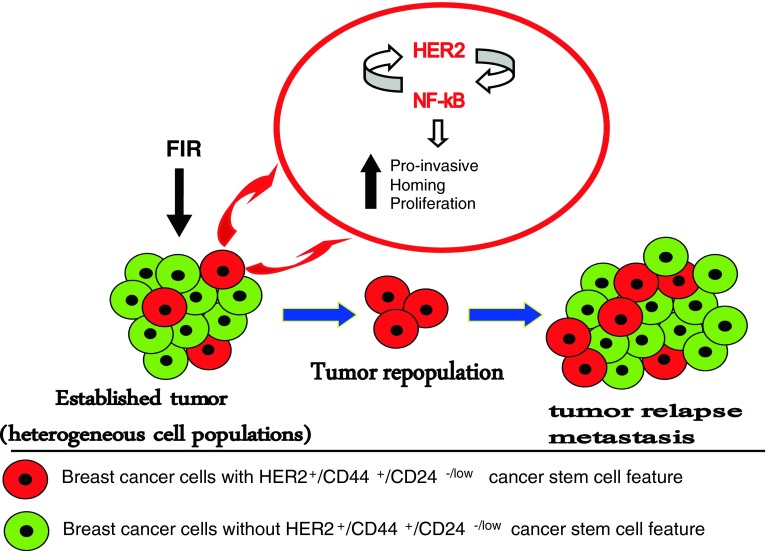
Schematic representation of HER2^+^/CD44^+^/CD24^−/low^, a new feature of breast cancer stem cells in tumor resistance to radiation. The radiation therapy applied to the established tumor composing from heterogeneous cell lines cause tumor regression. Breast cancer stem cells with CD44^+^/CD24^−/low^ marker and HER2 overexpression would be resistant to radiation therapy by activating HER2-NF-κB-HER2 signaling loop. The overexpression of both HER2 and NF-κB might change the gene profile of the cells with HER^+^/CD44^+^/CD24^−/low^ feature causing an increase in the number of genes responsible for proliferation, homing, and invasion. Thus, the initial regression in the tumor seen after radiation therapy would be because of the death of the cells without HER^+^/CD44^+^/CD24^−/low^ feature lacking activated HER2-NF-κB-HER2 loop. The selected cells with the HER^+^/CD44^+^/CD24^−/low^ feature, having stem cell-like properties, would self-renew and also give rise to the other cell types, making the tumor more aggressive and invasive

**Fig. 2:**
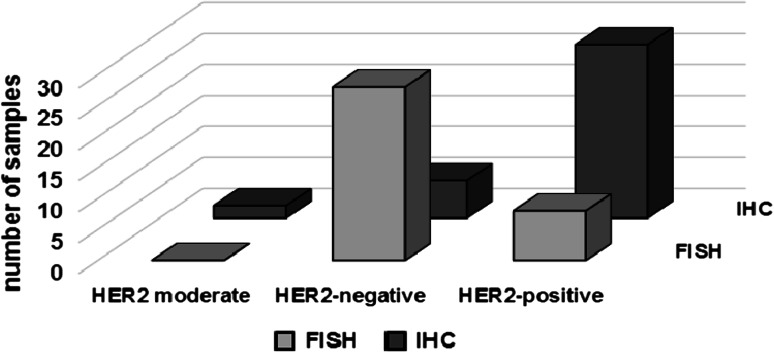
The comparison of the detection methods for confirming HER2 status of breast tumors. Several clinical trials show the discrepancy between the results obtained via FISH and IHC (adapted from Duru et al. 2012)

Data from our laboratory suggested HER2 as a marker for radioresistant breast CSCs as we have shown that HER2-expressing breast CSCs are more radioresistant and aggressive compared to HER2-negative breast CSCs (Cao et al. 2009; Duru et al. 2012). Thus, HER2-negative cancer may awake the silenced HER2 expression upon radiotherapy, and HER2-expressing breast CSCs are enriched in recurrent breast tumors (Figs. 1 and 2), which explains the potential clinical benefits of anti-HER2 therapy in originally HER2-negative breast cancer. Also, overexpression of HER2/neu in HER2-negative breast cancer cells resulted in resistance to anti-cancer radiation therapy via the activation of signaling pathways that involve NF-κB signaling (Cao et al. 2009). Although exact molecular networks causing radioadaptive tumor resistance in HER2-negative tumors are still largely unknown, data in our laboratory indicated that expression of HER2 is controlled by NF-κB, which is associated with FIR-induced radioresistance. With promoter/transcription factor analysis, potential NF-κB binding site was identified as GGG ACG ACC C (-364 bp to -355 bp) in HER2 promoter. Further, we found that NF-κB is recruited to HER2 promoter in vivo after radiation. When activation of NF-κB is inhibited, promoter activity of HER2 is also compromised. Importantly, NF-κB-mediated HER2 overexpression was found in FIR-induced radioresistant breast CSCs, which can be sensitized by a NF-κB inhibitor (Cao et al. 2009). Therefore, NF-κB-regulated HER2 signaling is critical for radiation-adaptive resistance in BCSCs. Finding the mechanisms underlying the adaptive radioresistance and identifying molecular markers of breast cancer stem cells holds great promise for effective cancer therapeutics. Given our recent findings that HER2-mediated radioresistance of breast cancer is initiated by radiation via NF-κB activation, blocking the NF-κB/HER2 network, including many mediators/effectors of the network, will be a crucial approach for sensitizing resistant breast cancer cells to radiotherapy.

## Activation of HER2-NF-κB-HER2 loop in breast cancer radioresistance

Overexpression of HER2 not only increases cell proliferation and survival (Kurokawa and Arteaga 2001), but also causes NF-κB activation via PI3 K/Akt pathway (Guo et al. 2004), which can be inhibited by the tumor suppressor phosphatase PTEN (Pianetti et al. 2001). Our laboratory previously reported that NF-κB and its regulated genes are activated by HER2 overexpression (Guo et al. 2004), and HER2-overexpressing MCF7/HER2 cells show enhanced resistance to IR-induced apoptosis with increased post-radiation clonogenic survival (Liang et al. 2003b; Pietras et al. 1999), while stable transfection of mutant IκB (MCF7/HER2/mIκB) or treatment with Herceptin inhibits NF-κB activation and radiosensitize them (Guo et al. 2004). We also found that Akt is required for HER2-mediated NF-κB activation in radiation response (Guo et al. 2004). Further studies showed that MAPK and PI3K/Akt pathways are involved in HER2-mediated resistance to radiation-induced apoptosis in breast cancer cells (Liang et al. 2003a), especially in HER2-expressing cells (Yang et al. 2000). Taken together, it is clear that NF-κB and HER2 are mutually dependent in signaling breast cancer radioresistance.

Breast cancer MCF7 cells that do not express a high level of HER2 can induce HER2 expression with adaptive radioresistance after exposure to fractionated high dose radiation (Guo et al. 2003; Li et al. 2001). Accumulating data suggest that there is a unique radioadaptive signaling pathway linked with induction of multidrug resistance-associated protein (MRP) (Harvie et al. 1997) and EGFR (Schmidt-Ullrich et al. 1997) in breast cancer cells. However, it has been recently shown that HER2 gene is also sensitive to radiation and can be induced by IR in breast cancer cell lines. IR-induced NF-κB mediates HER2 overexpression in the radioresistant breast cancer cells selected from FIR-derived heterogenic population (Cao et al. 2009), indicating that not only HER2 can induce NF-κB activity upon IR but also NF-κB can induce the overexpression of HER2. These results suggest a loop-like pathway of HER2-NF-κB-HER2 in tumor-adaptive radioresistance (Fig. 1). Our recent data support the hypothesis that HER2-NF-κB-HER2 loop is specifically conjugated with CD44^+^/CD24^−/low^ markers of breast CSCs (Duru et al. 2012). Identification of such positive loop of breast cancer radioresistance mediators may reveal the overly aggressive approach the cancer cells may take to achieve therapy resistance.

## Conclusion and perspective

In light of accumulating evidence of the features of CSCs in human cancer, the original BCSC biomarkers including CD44^+^/CD24^−/low^ should be revised to represent the increase in pro-invasiveness and aggressiveness since these two markers alone are not found to be sufficient for metastasis (Sheridan et al. 2006). The HER2-NF-κB-HER2 loop detected in radiation-treated breast cancer cells suggests that NF-κB and HER2 mediate each other’s expression (Cao et al. 2009), which is activated by anti-cancer therapy and thus a potential therapeutic target to sensitize breast cancer.

Another layer of tumor resistance is linked with the now-generally-acceptable cancer stem cell theory that tumor is a highly heterogeneous cell population. Both CSCs and non-CSCs are present where the population of the non-CSCs is higher than that of the CSCs, and the sensitivity of non-CSCs within the tumor is responsible for the initial tumor regression seen after radiation therapy. The CSCs are not killed because they activate the HER2-NF-κB-HER2 loop and, therefore, have selective advantage over non-CSCs against radiation (Fig. 1) (Duru et al. 2012). This hypothesis is supported by the clinical data, which show that breast cancer patients with recurrent invasive tumors are HER2-positive; however, the gene copy number of HER2 detected with FISH in the same patients is not elevated compared to the primary tumors (Cao et al. 2009). Several clinical trials show the discrepancy between the results obtained from FISH and IHC (Fig. 2). We have shown that the breast cancer cells, which are either missing HER2 or have low HER2 expression before radiation therapy, may present a radioresistant phenotype after the treatment due to the increased expression of HER2 and/or the HER2-NF-κB-HER2 loop activation following IR. These data are promising and important as they offer HER2 protein levels but not the HER2 gene copy number as a marker for breast cancer recurrence and radioresistance. These data are also crucial since they suggest that HER2 levels in the breast cancer patients should be closely monitored during the course of the treatment as HER2 expression might drastically increase as the therapy progresses. Further clinical studies are in need to verify that HER2 protein (detectable by IHC) as well as HER2 gene copy number is a dynamic feature in cancer cells and responsible for recurrent or metastatic tumors.

**Fig. 3:**
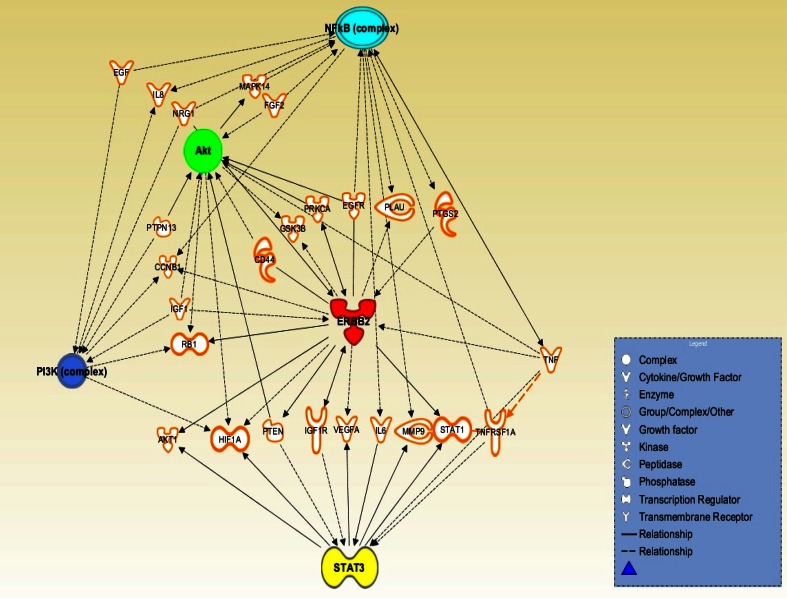
Schematic presentation of potential signaling network of HER2-NF-κB-PI3 K-Akt-STAT3. This signaling pathway is assumed based on the current literature, which, we believe, is activated by cellular adaptive response under anti-cancer chemo- and/or radio-therapy, and contributes to an adaptive resistance in cancer cells. Note that, the HER2-NF-κB-PI3 K-Akt-STAT3 pathway proposed here may be activated not only in the HER2-positive breast cancer but also activated in the HER2-negative cancer since *HER2* transcription is shown to be induced by radiation and DNA-damaging anti-cancer modalities without *HER2* gene copy enhancement

Further exploring the insight into HER2 network is also necessary. For instance, signal transducer and activator of transcription 3 (STAT3) in the HER2, NF-κB, PI3K/Akt network is promising since STAT3 emerges as another key player with an important role in HER2 positive breast CSCs (Fig. 3). HER2 is known to activate STAT3 through both JAK2- and Src-dependent manners (Ren and Schaefer 2002), and STAT3, in turn, promotes chemoresistance in head and neck cancer cells (Bourguignon et al. 2012). HER2-STAT3 signaling network is linked with the aggressiveness of HER2-expressing breast CSCs (Duru et al. 2012). Therefore, STAT3 may be the key factors downstream of HER2 activation (Korkaya et al. 2012). Moreover, it is known that STAT3 activates NF-κB, and its downstream targets Lin28 and let7 (Korkaya et al. 2011). Interestingly, in a recent paper, Lin28 expression was shown to be significantly associated with HER2 expression emphasizing that both proteins are associated with a poor clinical outcome for breast cancer (Feng et al. 2012). Lin28 can regulate HER2 such that Lin28 and HER2, two important stem cell regulatory genes, may act in a positive feedback loop (Malik et al. 2012). Therefore, investigation of the HER2-STAT3 signaling network and other HER2-linked effectors/pathways may provide more informative data on tumor adaptive resistance and effective targets to treat the aggressive recurrent and metastatic lesions.

## Abbreviations

AR: Acquired resistance
CSCs: Cancer stem cells
UPA: Urokinase plasminogen activator
ALDH1: Aldehyde dehydrogenase-1
RTKs: Receptor tyrosine kinases
AML: Acute myeloid leukemia
IR: Ionizing radiation
GAPDH: Glyceraldehydes-3-phosphate dehydrogenase
PGK1: Phosphoglycerate kinase 1
PTEN: Phosphatase and tensin homolog
Ras-MAPK: Ras-mitogen-activated protein kinase
MGMT: O^6^-Methylguanine-DNA methyltransferase
EGFR: Epidermal growth factor receptor
SirT1: Silencing information regulator2 homolog
HDAC: Histone deacetylase
ATM: Ataxia telangiectasia mutated
IKK: IκB kinase
NEMO: NF-κB essential modulator
SUMO: Small ubiquitin-like modifier
SH2: Src homology 2
PTB: Phosphotyrosine binding
PI3 K-PKB/Akt: Phosphatidylinositol 3′kinase-protein kinase B
PLC-PKC: Phospholipase C-protein kinase C
IHC: Immunohistochemistry
FISH: Fluorescence in situ hybridization
STAT3: Signal transducer and activator of transcription 3
